# Benign/Cancer Diagnostics Based on X-Ray Diffraction: Comparison of Data Analytics Approaches

**DOI:** 10.3390/cancers17101662

**Published:** 2025-05-14

**Authors:** Alexander Alekseev, Viacheslav Shcherbakov, Oleksii Avdieiev, Sergey A. Denisov, Viacheslav Kubytskyi, Benjamin Blinchevsky, Sasha Murokh, Ashkan Ajeer, Lois Adams, Charlene Greenwood, Keith Rogers, Louise J. Jones, Lev Mourokh, Pavel Lazarev

**Affiliations:** 1 Matur UK Ltd., 5 New Street Square, London EC4A 3TW, UK; aalexeev@matur.co.uk (A.A.); viacheslav.s@matur.co.uk (V.S.); oavdieiev@matur.co.uk (O.A.); plazarev@matur.co.uk (P.L.); 2 Department of Physics and Technology, Karaganda Buketov University, Karaganda 100028, Kazakhstan; 3 Institut de Chimie Physique, UMR8000, CNRS, Université Paris-Saclay, Bât. 349, 91405 Orsay, France; 4 Laboratoire de Physique des 2 Infinis Irène Joliot-Curie, UMR9012, CNRS, Université Paris-Saclay, Bât. 209, 91405 Orsay, France; 5 Stuyvesant High School, 345 Chambers Street, New York, NY 10282, USA; 6 School of Chemical and Physical Sciences, Keele University, Keele ST5 5BG, UKc.e.greenwood@keele.ac.uk (C.G.); 7 EosDx, Inc., 1455 Adams Drive, Menlo Park, CA 94025, USA; 8 Shrivenham Campus, Cranfield University, Swindon SN6 8LA, UK; k.d.rogers@cranfield.ac.uk; 9 Barts Cancer Institute, Queen Mary University of London, Charterhouse Square, London EC1M 6BQ, UK; l.j.jones@qmul.ac.uk; 10 Physics Department, Queens College, City University of New York, 65-30 Kissena Blvd., Flushing, NY 11367, USA

**Keywords:** structural biomarkers, X-ray diffraction, breast cancer diagnostics, machine learning, Fourier transformation

## Abstract

**Background/Objectives**: With the number of detected breast cancer cases growing every year, there is a need to augment histopathological analysis with fast preliminary screening. We examine the feasibility of using X-ray diffraction measurements for this purpose. **Methods:** In this work, we obtained more than 6000 diffraction patterns from 211 patients and examined both standard and custom-developed methods, including Fourier coefficient analysis, for their interpretation. Various preprocessing steps and machine learning classifiers were compared to determine the optimal combination. **Results:** We demonstrated that benign and cancerous clusters are well separated, with specificity and sensitivity exceeding 0.9. For wide-angle scattering, the two-dimensional Fourier method is superior, while for small angles, the conventional analysis based on azimuthal integration of the images provides similar metrics. **Conclusions**: X-ray diffraction of biopsy tissues, supported by machine learning approaches to data analytics, can be an essential tool for pathological services. The method is rapid and inexpensive, providing excellent metrics for benign/cancer classification.

## Simple Summary

Breast cancer is the most frequent cancer among women. Currently, histopathological analysis of biopsies is performed for both malignant and benign samples, with no effective triage system to ‘fast-track’ potential malignant cases. We propose a complementary method of benign/cancer classification based on X-ray scattering. Using and comparing machine learning approaches, we examined over 6000 measurements of benign and cancerous samples from 211 patients, achieving excellent results in distinguishing malignant and benign conditions. This can lead to a significant reduction in the turnaround time for the histopathological analysis and earlier diagnostics of malignancy, with potential impact on the survival rate for breast cancer patients.

## 1. Introduction

Breast cancer is the most commonly diagnosed cancer among women worldwide. The Lancet Breast Cancer Commission [1] predicts that by 2040, the global incidence of new cases of breast cancer will be more than 3 million per year. In the UK alone, there are 56,800 new cases of breast cancer diagnosed each year, with 11,500 deaths annually [2]. The mortality rate has started to decrease recently [3], which can be attributed to progress in early diagnostics [4,5]. Any delay in cancer detection can be fatal for a patient. All cancers currently require a histopathological examination, but such a procedure also expects the exclusion of benign lesions. Across most services, biopsies with a benign diagnosis are eight times more likely than those with a malignant one. Regardless, all require the same input level, and currently, there is no effective triage system to ‘fast-track’ potential malignant cases. The diagnostic problem being addressed by this work presents itself as a significant research question, i.e., can new diagnostic methods be developed and introduced into clinical pathways to effectively reduce the interval between clinical presentation and diagnosis?

In an average NHS Trust, there are about 60,000 breast biopsies per year, the majority of which will be benign. This places an enormous burden on Pathology Departments, where only 3% of laboratories are currently fully staffed [6], contributing to significant delays in turnaround time (TAT). Thus, there is a real need to adopt a different approach to tissue diagnostics that can effectively triage samples at an early stage, allowing those with potentially life-threatening conditions to be fast-tracked and, ideally, offering early reassurance to those without significant diseases. This is a component of the diagnostic space that our work is addressing. The problem of minimizing presentation-to-diagnosis time in order to provide improved outcomes is ultimately the issue that the work focuses on. Our solution is a novel diagnostic probe that can deliver significantly enhanced intelligence for clinicians. The work described herein is a step towards this goal.

To address this, we have investigated the feasibility of using the structural biomarkers obtained from X-ray diffraction to rapidly distinguish between cancer and benign breast tissues. Previously, cancer-induced structural changes were examined in two distinct ranges of scattering angles. Small-angle X-ray scattering (SAXS) and wide-angle X-ray scattering (WAXS) are defined to cover the (overlapping) momentum transfer values of 0.1 < *q* < 5 nm^−1^ and 3 < *q* < 45 nm^−1^, respectively. SAXS probes, for example, modifications to collagen fibril repeat distances [7,8,9,10], alterations to the amorphous scattering profile [9,10,11,12], and disruption to triglyceride molecular packing [13,14]. For the latter, a diffraction maximum at *q* = 1.5 nm^−1^ is characteristic of healthy tissue, and it has been shown [14] that this peak is absent in benign tissues. In contrast, WAXS addresses modifications to lipid and aqueous components [15,16,17,18,19,20,21]. Specifically, it has been shown [13,16] that in cancerous tissues, the intensity of a maximum peak at approximately *q* = 14 nm^−1^ is reduced, while the intensity maximum at approximately *q* = 20 nm^−1^ increases. The first feature is attributed to inter-fatty-acid molecular distances, while the second one is related to the oxygen–oxygen distance in the tetrahedral structure of water. The diminution of the 14 nm^−1^ peak is caused by cancer-altered lipid metabolism, rendering it a potential structural biomarker for cancer.

In the current study, we revisit and extend the measurements of the Breast Cancer Now Biobank (BCNB) [22] samples previously reported in [23] to specifically address the benign/cancer classification and determine the optimal preprocessing and classification methods. We utilize three distinct data representations, one of which is based on the analysis of one-dimensional (1D) momentum transfer profiles obtained after azimuthal integration. This representation was employed in [23]. Another approach was introduced in [24] for XRD-based cancer detection in dogs’ claws and implements the Fourier coefficients of this 1D profile. It was demonstrated in [25] for the same dataset of dogs’ claws that 2D Fourier analysis of the XRD images, without azimuthal integration, provides better metrics, which is our third data representation method. Various data preprocessing steps are employed, some of which are standard, including principal component analysis (PCA), and some are custom-developed. We use several machine learning classifiers, including Logistic Regression, Random Forest, Support Vector Machine, K-Nearest Neighbor, Naive Bayes Classifier, Light Gradient-Boosting Machine, and XGBoost. The first five classifiers are taken from the scikit-learn library [26,27], while the last two are acquired from [28] and the XGBoost library [29,30], respectively. The code is organized into pipelines, which describe different combinations of preprocessing, processing, and classification steps.

Our analysis shows excellent cancer/benign discrimination, with many pipelines exhibiting a balanced accuracy exceeding 0.9 for patients. In this, the obtained classification metrics are better for the WAXS images. A comparison of the data representation methods demonstrates that the 2D Fourier coefficients have an advantage, whereas, for 1D representation, the conventional approach is preferable to the 1D Fourier transform.

## 2. Materials and Methods

### 2.1. Experimental Design

#### 2.1.1. Breast Tissue Specimens

To test whether XRD could distinguish between cancer and benign tissue samples, we accessed ex vivo biopsy samples from the Breast Cancer Now Biobank (BCNB). Ethical approval for the project was obtained locally via Keele University (NS-210096) and for the collection and use of specimens via the BCNB (NRES Approval Number 23/EE/0229). These were fresh-frozen (FF) samples, approximately 10 × 2 × 2 mm in size, taken from patients who consented to the BCNB. All cases underwent full histopathological diagnosis on H&E sections and were categorized into invasive cancer or benign lesions. The cancer cohort mainly consisted of invasive ductal carcinoma and invasive lobular carcinoma of different grades. The benign cohort includes various conditions, excluding fibroadenomas and macromastia. The total numbers of patients, samples, and WAXS and SAXS measurements are presented in Table 1. The latter are almost identical, except one more cancerous sample was measured 9 times with SAXS, and one more benign measurement was conducted using WAXS. We obtained two samples from most patients, with a few providing one, three, or four samples. Depending on the size of the sample, it was measured in 4, 5, or 9 points to assess the heterogeneity.

Each tissue specimen was placed into a bespoke aluminum sample holder, 2 mm thick, utilizing a SPEX^TM^ 6 µm thick mylar window film to seal and secure the tissue within a 5 mm aperture.

#### 2.1.2. X-Ray Diffraction (XRD) Measurements

**Figure 1:**
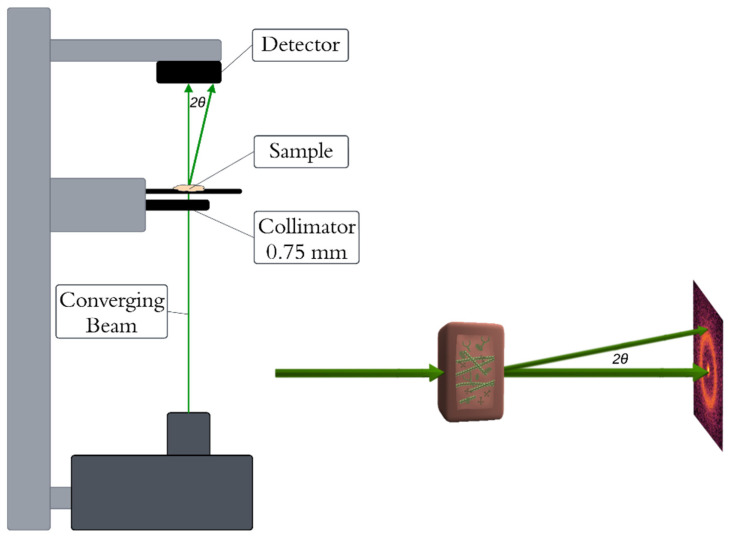
Schematic of the diffractometer.

XRD measurements were conducted using a bespoke X-ray diffractometer engineered and built by EosDx, Inc. (Menlo Park, CA, USA), a US-based company developing X-ray scattering for medical diagnostics. The schematic of the device is presented in Figure 1. The radiation produced by the copper-based Incoatec Microfocus Source (Geesthacht, Germany) was collected using two multilayer curved mirror optics, resulting in a low-divergence, monochromatic beam with a wavelength of *λ* = 0.154 nm. The two-dimensional detector employed to record the X-ray scatter was a MiniPix SN1442 Si 500 µm detector (ADVACAM, Prague, Czech Republic) with a 256 × 256-pixel array and a 55 × 55 µm pixel size.

**Figure 2:**
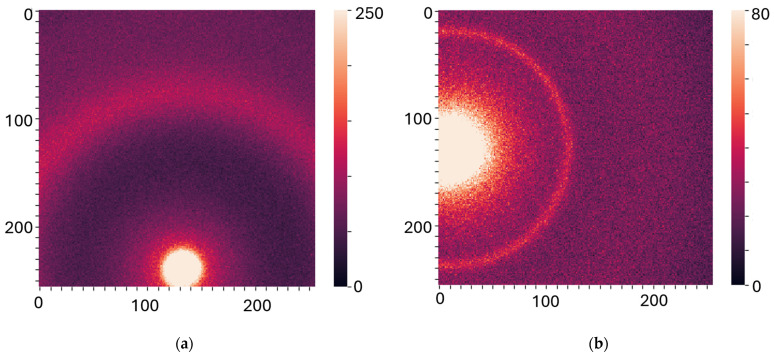
X-ray diffraction patterns for (**a**) WAXS and (**b**) SAXS. The labels on both axes are the pixel numbers.

Silver behenate powder (Thermoscientific^®^ 045494.06, Heysham, Lancashire, UK) was scanned (utilizing the bespoke aluminum holders) to allow for accurate sample-to-detector distance calibration. The experimental data were stored as a 256 by 256 matrix of integers representing the photon counts. All the experiments were performed at room temperature (19 °C) under atmospheric pressure. To assess specimen heterogeneity, data were collected from several individual spots on each sample, from 4 to 9, depending on the size of the sample, which resulted in several diffractograms per specimen. The measurements were performed at two specific sample-to-detector distances to examine WAXS (2 cm) and SAXS (16 cm) specifically. Each diffractogram was collected for 1 min for SAXS and 30 s for WAXS, with a count time of 0.1 s. Examples of the XRD images are shown in Figure 2a,b.

### 2.2. Data Analysis

#### 2.2.1. Image Preprocessing

In total, 3047 WAXS and 3055 SAXS images for 47 benign and 164 cancer patients were collected and examined in this work. The raw XRD data files contained the intensities in the 256 × 256-pixel arrays. In the first preprocessing step, any faulty detector pixels (e.g., dead pixels) were removed.

The maximum difference in sample-to-detector distance, which influences calibration, was within 2.6%. For the 1D models, which include azimuthal integration, automatic calibration was performed using the open-source library PyFAI. For 2D data representation, this error was considered negligible, and no size calibration was performed.

Additional preprocessing steps for the 2D models included normalizing the images outside the primary beam region. Furthermore, to exclude the contributions of random features, the areas outside of the main patterns, which were similar for all images, were nullified. The preprocessed images are presented in Supplemental Materials, Figure S1a,b.

**Figure 3:**
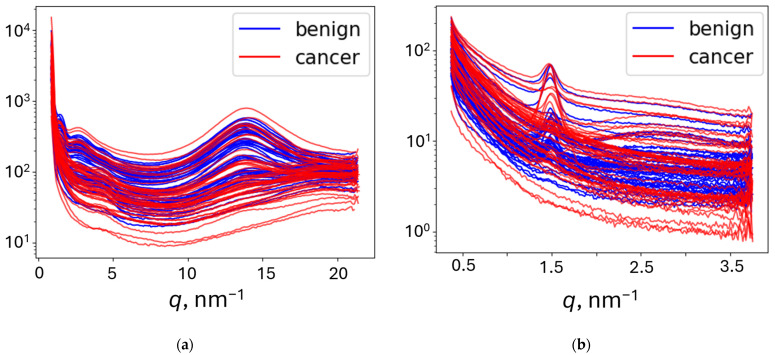
The intensity dependencies on the momentum transfer for benign and cancer samples, obtained after azimuthal integration of the XRD patterns measured at a (**a**) sample-to-detector distance of 2 cm (WAXS) and (**b**) sample-to-detector distance of 16 cm (SAXS).

For 1D models, the azimuthal integration was performed using PyFAI to obtain an average radial profile starting from the beam position. It was subsequently represented in terms of the momentum transfer *q* = (4π sin *θ*)/*λ*, where 2*θ* is the scattering angle, tan 2*θ* is calculated as the ratio of the distances from the pixel to the beam center and from the sample to the detector, and *λ* is the X-ray wavelength. To eliminate artifacts, the full *q*-range was limited to retain 3 ÷ 20 nm^−1^ for WAXS and 0.3 ÷ 3.7 nm^−1^ for SAXS. The obtained intensities were normalized. The representative curves are shown in Figure 3.

#### 2.2.2. Fourier Coefficient Representation

Both 1D and 2D Fourier transformations were implemented to describe the initial XRD data. The 1D Fourier coefficients were calculated for the azimuthally integrated (*AzI*) curves in Figure 2 using either the Discrete Fourier Transformation implemented in the SciPy and NumPy libraries (*1DF*) or the custom procedure described in [23] (*1DFC*). The slope of the curve was removed for improved Fourier series convergence (*SR*).

The 2D Fourier coefficients (*2DF*) were calculated for the preprocessed XRD data (Figure S1a,b) using the two-dimensional Discrete Fourier Transformation functions provided by the SciPy and NumPy libraries. This implemented preprocessing enabled analysis of the same area in 2D images, thereby enhancing the influence of cancer on the XRD pattern and reducing the impact of optical alignment. We tested different combinations of Fourier coefficient components: real parts (*Re*), imaginary parts (*Im*), amplitudes (*Am*), and phases (*Ph*).

To eliminate artifacts and accelerate processing, for some pipelines, we employed Low-Pass Fourier Filtration (LPF), which removed high-frequency coefficients. Specifically, for discriminant analysis, 30 coefficients were selected for each curve in the 1D case, and a 20th-order cutoff was implemented in the 2D case to retain only 1257 out of the total 65,536 coefficients.

#### 2.2.3. Measurements-to-Patients Transition

**Figure 4:**
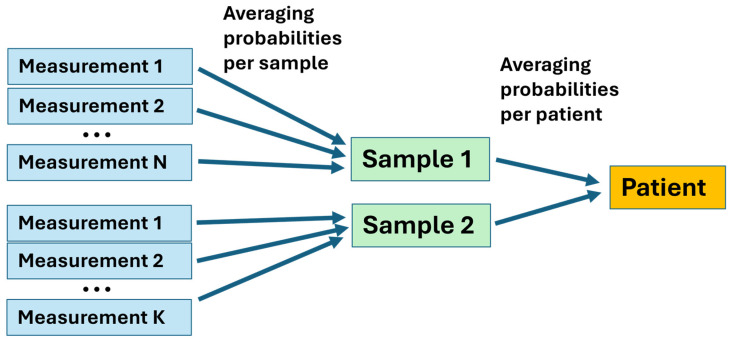
The measurements-to-patients transition procedure.

We examined two samples from most patients, with a few providing one, three, or four samples, and each sample was measured multiple times. The model was optimized using all measurements in the training dataset. Then, the class probability of each testing measurement was evaluated using the optimized model, which provided the decision threshold to achieve maximum balanced accuracy. Cancer and benign diagnoses were assigned for each measurement when the probability exceeded or fell below the threshold, respectively. The final diagnosis of a patient was established by averaging all predicted class probabilities for that patient and comparing them with the optimal decision threshold (Figure 4).

#### 2.2.4. Data Analysis Procedure and Machine Learning Methods

We used Visual Studio (version 1.97.2) and Jupyter Notebook (version 7.2.2) as the primary coding platforms. The majority of preprocessing methods were sourced from the scikit-learn library [27], from which most classifiers were also adopted, except for gradient-boosting algorithms [28,29,30]. Here, we provide only a brief description of the computation method, as the overall classification algorithm is the same as described in [25], where all details can be found. All preprocessing steps are optional, and they were combined differently in the pipelines. Abbreviations given here in italics will be used in the Results section to describe the pipelines. Optionally, the standardization (*STD*) of Fourier coefficients can be applied before classification. As a dimensionality reduction technique, principal component analysis (PCA) was used with three different numbers of principal components: 3 (*PCA_3*), 50 (*PCA_50*), and 100 (*PCA_100*). Two methods for removing the primary beam were implemented in the code. The beam was removed from the original images before the Fourier transformations (*BR*) and, alternatively, in reciprocal space (*BRF*) (see [25] for details).

**Figure 5:**
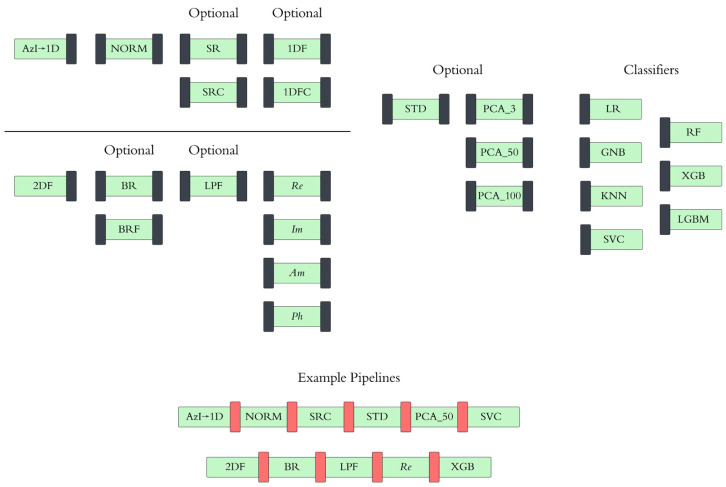
Schematic of the pipeline approach, including mandatory and optional steps, with two specific pipeline examples.

The code was organized into pipelines, which contained different combinations of preprocessing, processing, and classification steps. It should be emphasized that the values of the optimal decision threshold vary broadly between the pipelines. Some pipelines with too many features used in modeling require the Stochastic Gradient Descent (*SGD*) method [27] to ensure reasonable computation time. The list of classifiers used in this work included Logistic Regression (*LR*), Gaussian Naive Bayes (*GNB*), a K-Nearest Neighbor Classifier (*KNN*), a linear Support Vector Classifier (*SVC*), a Random Forest Classifier (*RF*), XGBoost (*XGB*), and LightGBM (*LGBM*). The pros and cons of each classifier were previously discussed in [25]. The schematic of the pipeline approach is presented in Figure 5.

For the 1D approach, we performed the azimuthal integration of the image, followed by normalization. Optionally, we can remove the slope and calculate the Fourier coefficient, using the standard or custom procedures. For 2D, we always performed the Fourier transformation, with or without one of the two types of beam removal, and an optional Low-Pass Filter. Various components of the Fourier coefficients (*Re*, *Im*, *Am*, or *Ph*) were used for the analysis. For both the 1D and 2D cases, PCA can be utilized (jointly with *STD*) with 3, 50, or 100 principal components. Finally, any of the seven classifiers can be employed to obtain the performance metrics. Two examples are shown in Figure 5. In the first one, azimuthal integration and normalization are followed by the custom slope removal and PCA with 50 components, with SVC as the classifier. In the second example, the beam is removed in the real space, the LPF cuts out a number of the Fourier coefficients, and their real parts are examined by the XGB classifier.

In total, we produced 574 pipelines, both for WAXS and SAXS, divided into three main types: 1D models with Fourier transformation (*1DF*) and without it (*1D*), as well as 2D models with Fourier transformation (*2DF*). A complete list of abbreviations for the utilized methods is provided in Supplemental Table S1.

## 3. Results

The measured XRD patterns were randomly separated into training and testing datasets, with 20 cancerous (1–20) and 12 benign (21–32) patients selected for testing. After both the training and testing datasets were preprocessed using the procedure described above, the training dataset was used to optimize the model. The testing dataset was then classified based on the optimized estimator.

We used sensitivity (Sen_M), specificity (Spec_M), and balanced accuracy (BA_M) as performance metrics to assess the measurements. Sensitivity and specificity are the proportions of cancerous and benign samples, respectively, that were correctly identified, and balanced accuracy is the average of these two metrics. We also determined the receiver operating characteristic (ROC) curve and used the area under the ROC curve (AUC_M) as a metric. After the transition to patients described above, all performance metrics (Sen_P, Spec_P, BA_P, and AUC_P) were also calculated for the patients. All the procedures were performed separately for the WAXS and SAXS measurements.

The pipelines, i.e., combinations of preprocessing steps and classifiers, were ranked in terms of BA_P and AUC_P (for equal BA_P values). The five highest-ranked sets are presented in Table 2 and Table 3 for WAXS and SAXS, respectively. The total rankings for WAXS (W) and SAXS (S) are provided in the first column. The second column describes the set of preprocessing steps and the classifier using the nomenclature introduced in Section 2. Columns 3–6 show the metrics for the measurements, and columns 7–10 display the metrics for patients.

It is evident that many pipelines provided excellent metrics, demonstrating a clear separation of the diffraction patterns belonging to cancerous and benign tissues. For WAXS, the most discriminating results are for the two-dimensional analysis. However, the metrics for the 1D approach are also reasonably good. The best 1D pipelines based on the 1D Fourier coefficients are ranked 84W–92W, with identical BA_P = 0.91 and AUC_P = 0.9–0.93 for the RF, XGB, KNN, and LGBM classifiers. The conventional 1D analysis is ranked higher, with 43W–48W, yielding BA_P = 0.93 and AUC_P = 0.9–0.93 for the RF, XGB, KNN, SVC, and LGBM classifiers. The LR classifier produces an even higher value, with AUC_P = 0.97. In general, BA_P for 99 out of 574 pipelines exceeds 0.9. For SAXS, the metrics for 2D and conventional 1D are similar, while the best 1D Fourier is ranked 25S, with BA_P = 0.88 and AUC_P = 0.86 for the LR classifier. The results for SAXS are worse than those for WAXS, with only 12 pipelines providing BA_P better than 0.9.

In the analysis of WAXS, the metrics for all classifiers are similar, except for GNB, which showed impressive results only when combined with PCA. It is worth noting that this is the only classifier to perform better with PCA, while all the others showed no improvement. For SAXS, LR and SVC perform better than the others, while KNN performs worse, with metrics sometimes improved by PCA.

**Figure 6:**
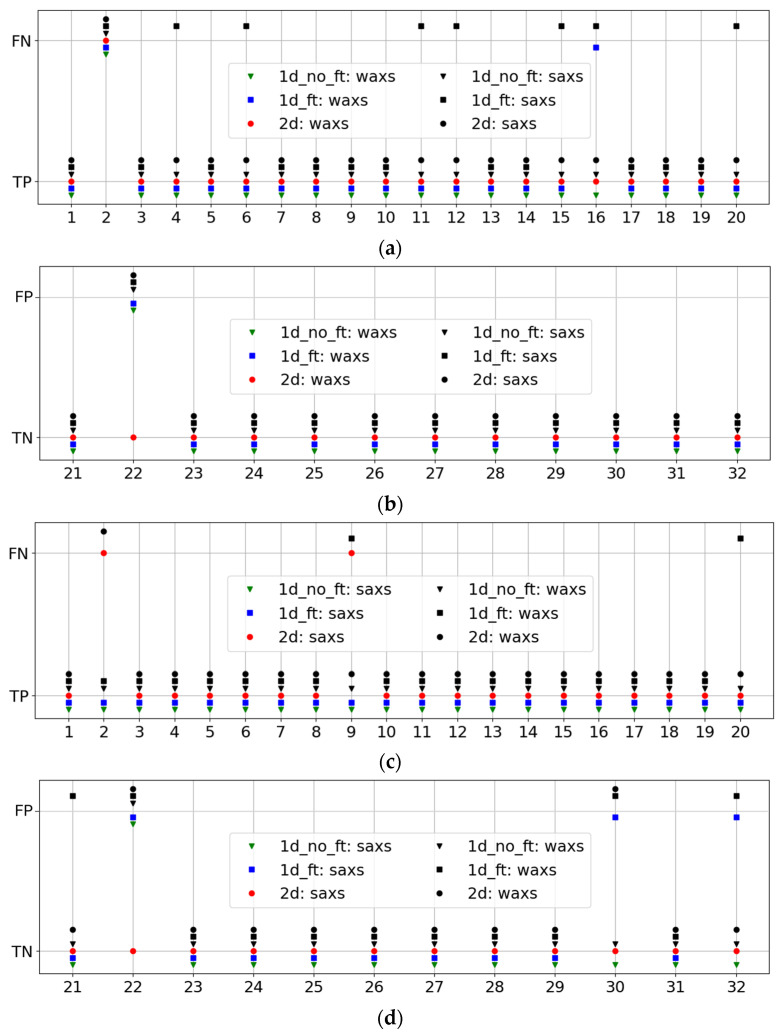
Performances of pipelines with best metrics for patients in (**a**) WAXS cancer, (**b**) WAXS benign, (**c**) SAXS cancer, and (**d**) SAXS benign. Cancer patients are numbered from 1 to 20, and benign patients are numbered from 21 to 32. FN, FP, TN, and TP indicate false negative, false positive, true negative, and true positive, respectively.

To determine the causes of our <1 metrics, we examined the results of the best pipelines for all patients from the testing group. The resulting performances are displayed in Figure 6 for WAXS (a,b) and SAXS (c,d). In this, we also demonstrate the indirect performances of the best WAXS (SAXS) pipelines of all three types on SAXS (WAXS) datasets. They are shown in black, while the best direct ones are in green, blue, and red, depending on the pipeline type.

It is evident from this figure that pipelines with excellent performance on WAXS are not always reliable for the SAXS dataset, and vice versa. A good illustration is Figure 6a, which shows many false-negative results (black squares) for the indirect 1DF pipeline.

This analysis revealed two outstanding patients: Patient 2, with a cancer diagnosis, and Patient 22, with benign conditions. For Patient 2, all pipelines yielded false negatives in WAXS, as did the 2D pipelines in SAXS. For Patient 22, all pipelines, except the direct 2D pipeline, resulted in a false positive. New histopathological analyses were performed for these patients. For Patient 22, a fibroadenoma diagnosis was obtained, which explained the false-positive results, as fibroadenoma patients were not included in the benign training dataset. For Patient 2, the new tissue assessment described the tissue as only 5% invasive ductal carcinoma.

**Figure 7:**
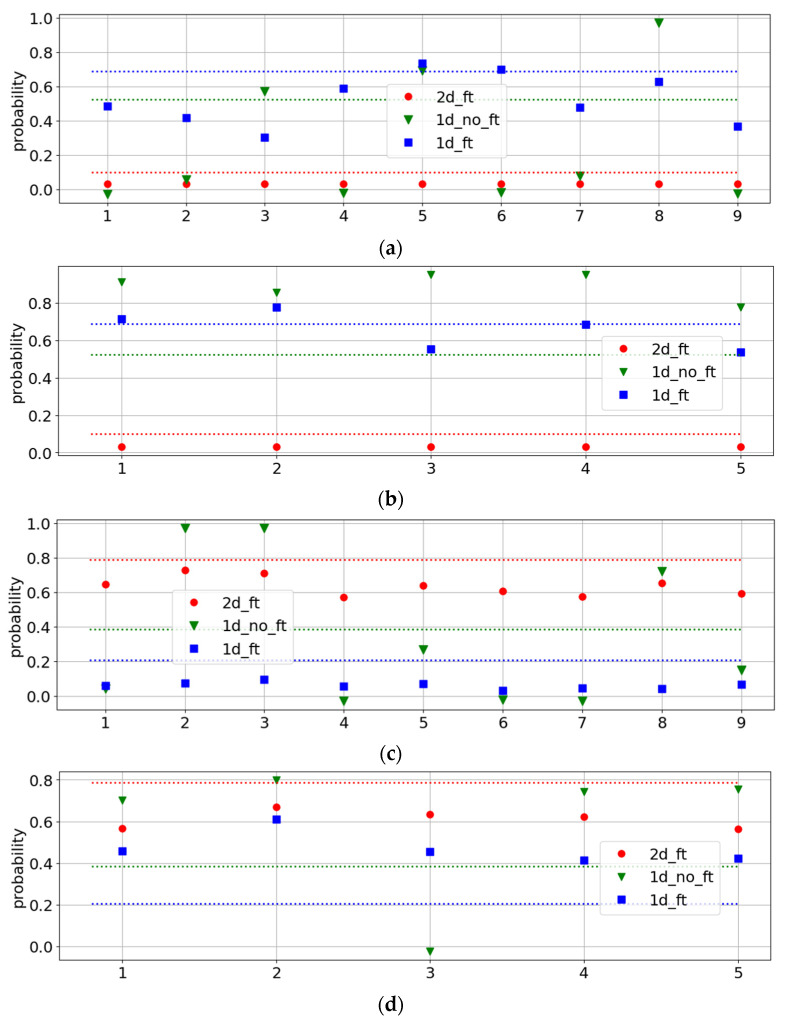
Performances of pipelines with best metrics for measurements of Patient 2 for (**a**) WAXS, sample 1, (**b**) WAXS, sample 2, (**c**) SAXS, sample 1, and (**d**) SAXS, sample 2. The horizontal lines are the optimal decision thresholds for the pipelines of the same color.

To gain a better understanding of Patient 2, we examined all the measurements of the corresponding samples, along with the class probabilities and optimal decision thresholds, as shown in Figure 7, for the best pipelines of all types.

The two samples belonging to Patient 2 exhibit different behaviors seen in the 1D pipelines, while for the 2D pipelines, they are similar. The first sample, measured at nine points, was closer to the benign response, while the second sample was rather cancerous. However, even in the first sample, the class probabilities for different measurements vary from 0 to 1 in the conventional 1D classification. This agrees with a new tissue assessment reporting that this patient had an initial or even pre-cancerous stage; the tumor microenvironment is not entirely restructured by the cancer cells, and the diffraction patterns strongly depend on the point of measurement. Such non-uniformity of the X-ray scattering is especially seen in the SAXS measurements.

## 4. Discussion

In this study, we measured X-ray diffraction patterns from 211 patients with benign and malignant histological diagnoses. The measurements were performed in two separate ranges of the scattering momentum transfer, varying the sample-to-detector distance of the diffractometer. We compared various data analytics methods with different sets of preprocessing steps and machine learning classifiers (pipelines). We achieved excellent benign/cancer classification, with more than a hundred pipelines having balanced accuracy exceeding 0.9. These results are significantly better than those previously obtained in [23] for part of the dataset. However, it is worth noting that for the present paper, we excluded the “healthy” samples derived from cosmetic procedures and benign samples with confirmed diagnoses of fibroadenoma and macromastia.

We compared three different data representations and demonstrated that the custom-developed approach, based on the 2D Fourier coefficients [25], yields better metrics for wide-angle scattering. For small angles, the results obtained by this approach are similar to those obtained by the conventional method based on azimuthal integration. At the same time, using the Fourier transformation for the examination of the 1D curves [24] does not appear to be beneficial, as the metrics are worse. The standard and custom-developed Fourier transformation procedures produced identical results. Generally, the classification performs better for WAXS measurements than for SAXS.

We also compared various machine learning classifiers and demonstrated that the results are comparable, with minor exceptions. One such exception is the performance of the Logistic Regression. In [25], where X-ray diffraction of dogs’ claws was examined, this classifier was worse than any of the others. Here, it provides improved metrics, especially in the SAXS range. A possible explanation is that cancer and benign clusters are well defined, in contrast to dogs’ claws, and the straightforward separation using Logistic Regression works well.

We also investigated the reasons why our analysis was non-ideal and identified two outliers in the test dataset, for which almost all pipelines produced either a false negative (for the cancer patient) or a false positive (for the benign patient). A new histopathological tissue assessment revealed that the benign patient had a fibroadenoma, which was an exclusion criterion for the training dataset; therefore, these data do not belong to either of the established clusters. For the data of the second anomalous patient, a similar new tissue assessment showed that this is only 5% invasive ductal carcinoma. A more scrutinized examination of the two samples belonging to this patient revealed the non-uniformity of results between the samples and even within different positions on the same sample. This is expected, as the cancer-induced structural changes have not yet extended to the macroscopic scale in such patients.

The revealed heterogeneity informs us regarding one of the future improvements of our method. In the present work, we employed simple averaging of the results from different measurements and samples, as shown in Figure 4. However, for non-conclusive cases, a more advanced weighted approach could be used. Another improvement can be the inclusion of healthy samples and patients with fibroadenoma and macromastia. In this case, the binary classification would be replaced with a multiclass classification, as several clusters are expected. However, we should emphasize that the present work is only the first step, and further research is necessary with greatly enhanced statistics.

## 5. Conclusions

Our results clearly showed that cancer-induced structural biomarkers can be successfully revealed by X-ray diffraction of human breast tissues. Our prototype measurement system features a variable sample-to-detector distance, enabling it to accommodate a range of momentum transfer values from the SAXS to the WAXS regions. Such measurements probe different independent molecular components of tissues and, therefore, complement each other.

In the present work, we developed and compared various data analytics approaches, which include both conventional 1D data representation based on the azimuthal integration of the diffraction patterns and 1D and 2D Fourier transformations. We obtained excellent performance metrics, especially with 2D Fourier coefficients in the WAXS range. Our measurements and data analysis are fast and inexpensive, making X-ray diffraction a valuable tool for pathological laboratories. Thus, this work makes a significant contribution to the steps required in the development and ultimate deployment of diffraction-based technologies to address the issue of minimizing presentation-to-diagnosis periods. In particular, it identifies optimal data reduction and analysis protocols. The work also provides valuable data and analysis approaches for other research groups studying the exploitation of X-ray scatter signatures for medical diagnostics.

## Figures and Tables

**Table 1:** Numbers of patients, samples, and measurements for WAXS/SAXS.

	Train			Test			Total
	Benign	Cancer	Total	Benign	Cancer	Total	
Patients	35	144	179	12	20	32	211
Samples	80	283/284	363/364	25	40	65	428/429
Measurements	623/622	1960/1969	2583/2591	208	256	464	3047/3055

**Table 2:** Five highest-ranked metrics for various preprocessing steps and classifiers for WAXS.

	Steps and Classifiers	Sen_M	Spec_M	AUC_M	BA_M	Sen_P	Spec_P	AUC_P	BA_P
1W	*2DF*, *BR* or *BRF*, *LPF*, *Re*, *LR*	0.54	0.99	0.77	0.78	0.95	1	0.97	0.975
2W	*2DF*, *BR*, *Am*, *LR*	0.85	0.82	0.91	0.83	1	0.92	0.95	0.96
3W	*2DF*, *BR*, *Am*, *XGB*	0.94	0.66	0.87	0.8	1	0.92	0.93	0.96
4W	*2DF*, *LPF*, *STD*, *Re*, *PCA_50*, *XGB*	0.95	0.74	0.9	0.845	0.95	0.92	0.96	0.935
5W	*2DF*, *BR* or *BRF*, *Re*, *XGB*	0.88	0.84	0.93	0.86	0.95	0.92	0.95	0.935

**Table 3:** Five highest-ranked metrics for various preprocessing steps and classifiers for SAXS.

	Steps and Classifiers	Sen_M	Spec_M	AUC_M	BA_M	Sen_P	Spec_P	AUC_P	BA_P
1S	*1D*, *STD*, *LR*	0.8	0.91	0.905	0.855	1	0.92	0.95	0.96
2S	*2DF*, *BR* or *BRF*, *LPF*, *Re*, *SVC*	0.92	0.76	0.91	0.84	0.9	1	0.97	0.95
3S	*1D*, *STD*, *SVC*	0.92	0.88	0.935	0.9	0.95	0.92	0.95	0.935
4S	*2DF*, *BR*, *Am*, *SVC*	0.76	0.87	0.89	0.815	0.9	0.92	0.92	0.91
5S	*1D*, *STD*, *PCA_3*, *SVC*	0.8	0.92	0.89	0.86	0.9	0.92	0.915	0.91

## Data Availability

The files with the XRD patterns for the training and test datasets, as well as the complete tables with sets of metrics obtained from different approaches, are available at https://zenodo.org/records/15129858 (published 3 April 2025). The codes are available upon request.

## Supplementary Materials

The following supporting information can be downloaded at https://www.mdpi.com/article/10.3390/cancers17101662/s1: Table S1: List of abbreviations; Figure S1: The pre-processed images: (a) WAXS and (b) SAXS.
