# Supplementary material for: Benign/Cancer Diagnostics Based on X-Ray Diffraction: Comparison of Data Analytics Approaches
Source: Cancers (Basel). 2025 May 14;17(10):1662. doi: 10.3390/cancers17101662 (PMC12109960; doi:10.3390/cancers17101662)
Supplement: Supplementary file 1 [file cancers-17-01662-s001.zip › Table S1. List of abbreviations.pdf]

1DF	1-Dimensional Fourier coefficients calculated through SciPy and NumPy
1DFC	1-Dimensional Fourier coefficients calculated through the Custom procedure
2DF	2-Dimensional Fourier coefficients
Am	Amplitudes of Fourier coefficients
AUC_M	Area Under ROC Curve for Measurements
AUC_P	Area Under ROC Curve for Patients
BA_M	Balanced Accuracy for Measurements
BA_P	Balanced Accuracy for Patients
BCNB	Breast Cancer Now Biobank
BR	Beam Removal in real space before the Fourier transformations
BRF	Bean Removal in reciprocal space
FF samples	Fresh-Frozen samples
GNB	Gaussian Naive Bayes Classifier
Im	Imaginary parts of Fourier coefficients
KNN	K-nearest Neighbors Classifier
LGBM	LightGBM Classifier
LPF	Low-Pass Fourier Filtration
LR	Logistic Regression Classifier
PCA	Principal Component Analysis
PCA_100	Principal Component Analysis with 100 components
PCA_3	Principal Component Analysis with 3 components
PCA_50	Principal Component Analysis with 50 components
Ph	Phases of Fourier coefficients
Re	Real parts of Fourier coefficients
Re_Im	Both Real and Imaginary parts
RF	Random Forest Classifier
ROC curve	Receiver Operating Characteristics curve
SAXS	Small-Angle X-ray Scattering
Sen_M	Sensitivity for Measurements
Sen_P	Sensitivity for Patients
SGD	Stochastic Gradient Descent
Spec_M	Specificity for Measurements
Spec_P	Specificity for Patients
SR	Slope Removal
STD	Standardization of Fourier Coefficient
SVC	Linear Support Vector Classifier
WAXS	Wide-Angle X-ray Scattering
XGB	XGBoost Classifier
XRD	X-ray Diffraction
