# Supplementary figures and images for: Benign/Cancer Diagnostics Based on X-Ray Diffraction: Comparison of Data Analytics Approaches
Source: Cancers (Basel). 2025 May 14;17(10):1662. doi: 10.3390/cancers17101662 (PMC12109960; doi:10.3390/cancers17101662)

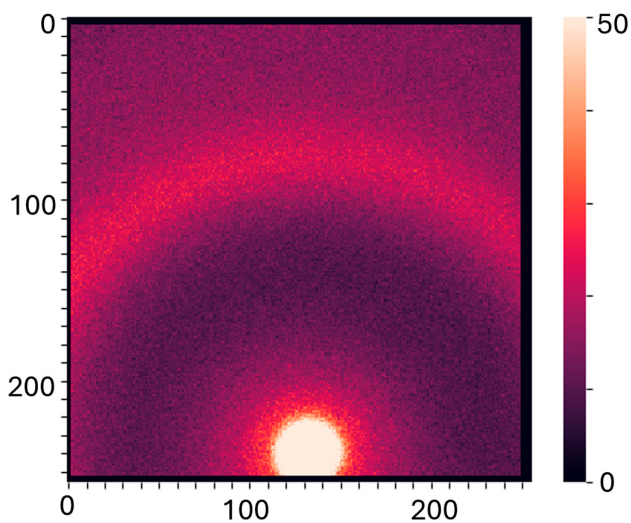

(a)

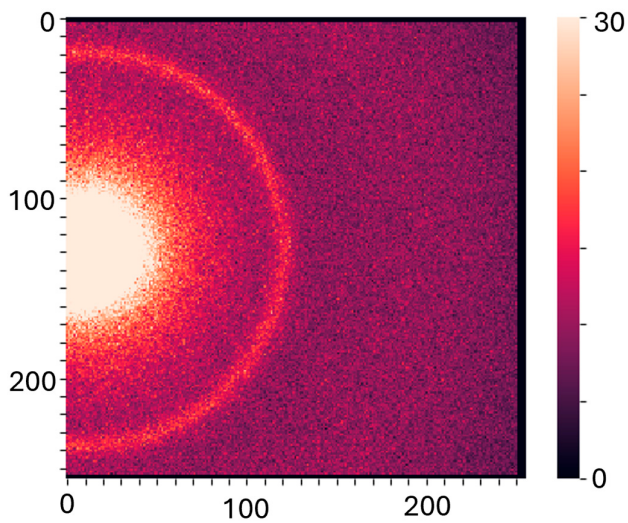

(b)

**Figure S1.** The pre-processed images: (a) WAXS and (b) SAXS.

Supplement: Supplementary file 1 [file cancers-17-01662-s001.zip › Figure S1.pdf]
